# Selection of patients with symptomatic vagal-induced sinus node dysfunction: Who will be the best candidate for cardioneuroablation?

**DOI:** 10.3389/fphys.2023.1088881

**Published:** 2023-02-07

**Authors:** Simin Cai, Lihui Zheng, Yan Yao

**Affiliations:** ^1^ Cardiac Arrhythmia Center, Heart Center, The People’s Hospital of Zhengzhou University, Henan Provincial People’s Hospital, Huazhong Fuwai Hospital, Zhengzhou, Henan, China; ^2^ Cardiac Arrhythmia Center, National Center for Cardiovascular Diseases, Chinese Academy of Medical Sciences and Peking Union Medical College, Fuwai Hospital, Beijing, China

**Keywords:** cardioneuroablation, sinus node dysfunction, selection criteria, cardiac vagal tone, deceleration capacity of heart rate

## Abstract

Sinus node dysfunction is a multifaceted disorder with variable manifestations, the prevalence of which increases with age. In a specific group of patients, excessive vagal activity may be the sole cause for this condition. These patients are characterized as having recurrent daytime symptoms attributed to bradyarrhythmia, no evidence of organic sinus node lesions, cardiac vagal overactivation, and are non-elderly. For sinus node dysfunction patients, a permanent pacemaker implantation appears to be the ultimate solution, although it is not an etiological treatment. Cardioneuroablation is a promising emerging therapy that can fundamentally eliminate symptoms in a highly selective sub-set of sinus node dysfunction patients by cardiac vagal nerve denervation. Denervation with ablation for vagal-induced sinus node dysfunction can effectively improve sinus bradycardia and reduce syncope. To date, guidelines for selection of suitable candidates for cardioneuroablation remain lacking. The primary objective of this study was to distinguish the nature of abnormal sinus node function and to find methods for quantifying vagal tone. Clear selection criteria could help physicians in identification of patients with autonomic imbalance, thereby maximizing patient benefits and the success rate of cardioneuroablations.

## 1 Introduction

Cardiac physiological function is regulated by the epicardial autonomic ganglia of the heart. Cardiac innervation of the autonomic nervous system converges at multiple ganglionated plexi (GPs) that contain sympathetic and parasympathetic neurons (Armour et al., 1997; Chiou et al., 1997). Autonomic perturbation of parasympathetic overactivity may induce the onset of vasovagal syncope (VVS), functional sinus node dysfunction (SND), and functional atrioventricular block (FAVB) (Manolis et al., 2021). These patients are at a high risk of injury and severe impacts on quality of life if undergone hemodynamic disturbance, may be refractory to conventional therapies, and require pacemaker implantation to prevent sudden fatal events (Kusumoto et al., 2019; Glikson et al., 2021).

Cardioneuroablation (CNA) is an emerging interventional therapy that localizes vagal GPs *via* electroanatomical mapping and damages nerve fibers *via* radiofrequency ablation. It can blunt the excessive vagal activation of the heart and thus eliminate or improve the pathological conditions related to high vagal tone (Pachon et al., 2005). CNA has been used successfully in preliminary studies in refractory VVS patients, particularly in treating the cardioinhibitory type (significant decrease in heart rate during syncope, occasionally accompanied with paroxysmal abnormal rhythm such as sinus bradycardia, sinus arrest, and atrioventricular block), and may enable patients to avoid the complications and financial burdens of long-term cardiac pacing (Pachon et al., 2005; Pachon et al., 2011; Yao et al., 2012; Aksu et al., 2016; Debruyne et al., 2018; Hu et al., 2019; Aksu et al., 2020; Pachon et al., 2020; Aksu et al., 2021; Debruyne et al., 2021; Tu et al., 2022).

Previous studies exploring the therapeutic effects of CNA in vagal-induced bradycardia and conduction disorders have suggested CNA as an alternative treatment strategy for patients with functional SND and FAVB after pharmacological interventions have failed (Pachon et al., 2005; Zhao et al., 2015; Aksu et al., 2016; Qin et al., 2017; Debruyne et al., 2018; Aksu et al., 2020; Aksu et al., 2021; Debruyne et al., 2021). SND encompasses a range of electrophysiological and arrhythmic disturbances resulting from abnormalities of the sinus node and atrial impulse formation and propagation (Kusumoto et al., 2019).

The clinical manifestations of SND can vary from insidious symptoms to a state of frequent discomfort such as a constant feeling of fatigue or recurrent syncope (Kusumoto et al., 2019). Among the extrinsic factors (autonomic perturbation, metabolism, and medication) affecting sinus node function, vagal overactivity may play the most important role (Kusumoto et al., 2019); in this review, functional SND refers to vagally induced SND. Although symptomatic SND is an indication for pacemaker implantation, the premise is that other potential treatable or reversible etiologies have been excluded (Kusumoto et al., 2019; Glikson et al., 2021). Determining whether an abnormal heart rhythm is caused by the relatively reversible autonomic imbalance, that is, distinguishing between symptom-related vagal overactivity and sinus node intrinsic damage accurately, is the focus and difficulty of rational and effective application of CNA in SND patients. Thus, when making therapeutic decisions for SND patients, in addition to the need to obtain a clear symptom-rhythm correlation, quantifying the cardiac vagal tone and clarifying the nature of sinus node lesions are equally important. In this review, we provide an overview of the knowledge regarding symptomatic vagal-induced SND, therapy choices for refractory patients, and the strengths and limitations of different diagnostic tools for evaluating the function of the sinus node or cardiac autonomic nervous system.

## 2 Cardiac autonomic nervous system

The sympathetic and parasympathetic nervous systems, the two core arms of the autonomic nervous system, regulate the basic physiological activities of any visceral organ, and interact at each hierarchical level, including brain centers, brainstem, spinal cord, stellate ganglia, and intrinsic visceral nerves and ganglia (Manolis et al., 2021). The cardiac autonomic nervous system is also divided into two parts: the extrinsic part, derived from the central nervous system and its emanated axons, mediates connections between the heart and the autonomic ganglia of the cervical and thoracic spinal cord (sympathetic connections) and the medulla oblongata (parasympathetic connections, the vagus nerve carrying parasympathetic preganglionic fibers); the intrinsic part is located in the periatrial epicardial fat pads that contain the autonomic ganglia and the downstream autonomic nerve fibers, mainly innervating the sinus node, atrial muscle, atrioventricular node, and ventricular muscle. Besides acting as a relay-station that processes efferent signals to the heart, it is also involved in the local reflex loop that regulates cardiac function independently of the control of higher autonomic centers (Armour et al., 1997; Chiou et al., 1997; Manolis et al., 2021). Cardiac motor neurons exert their effect *via* a fine balance between cholinergic (parasympathetic postganglionic nerve fibers) and adrenergic (sympathetic postganglionic nerve fibers) fibers, the former cause negative chronotropic and dromotropic effects through releasing acetylcholine (ACh), while the latter enhance cardiac contractility and accelerate myocardial electrical conduction by releasing norepinephrine (Shivkumar et al., 2016; Manolis et al., 2021). The cardiac autonomic nervous imbalance is one of the etiologies for the genesis of arrhythmia, such as bradycardia (Manolis et al., 2021).

## 3 Sinus node dysfunction with vagal overactivity

SND is linked to impaired pacemaker function in the sinus nodes with various presentations, including sinus bradycardia (sinus rate <50 beats per minute), sinus pause (depolarization ceases >3 s) or arrest, sinoatrial exit block, ectopic atrial bradycardia, alternating periods of atrial tachyarrhythmias and bradyarrhythmias (tachycardia-bradycardia syndrome), inappropriate variation in heart rate during stress or exercise (chronotropic incompetence), and isorhythmic dissociation (Kusumoto et al., 2019). Patients usually have accompanying symptoms (dizziness, amaurosis, fatigue, and palpitation), and syncope; even sudden death has been noted in severe cases (Kusumoto et al., 2019). Syncope is a common complaint in patients with SND and has been reported in 50% of patients undergoing pacing therapy (Nielsen et al., 2011). These symptoms may occur at rest, during or after exercise, and in a pathological pause at the end of an episode of atrial tachyarrhythmia (Dobrzynski et al., 2007).

**FIGURE 1:**
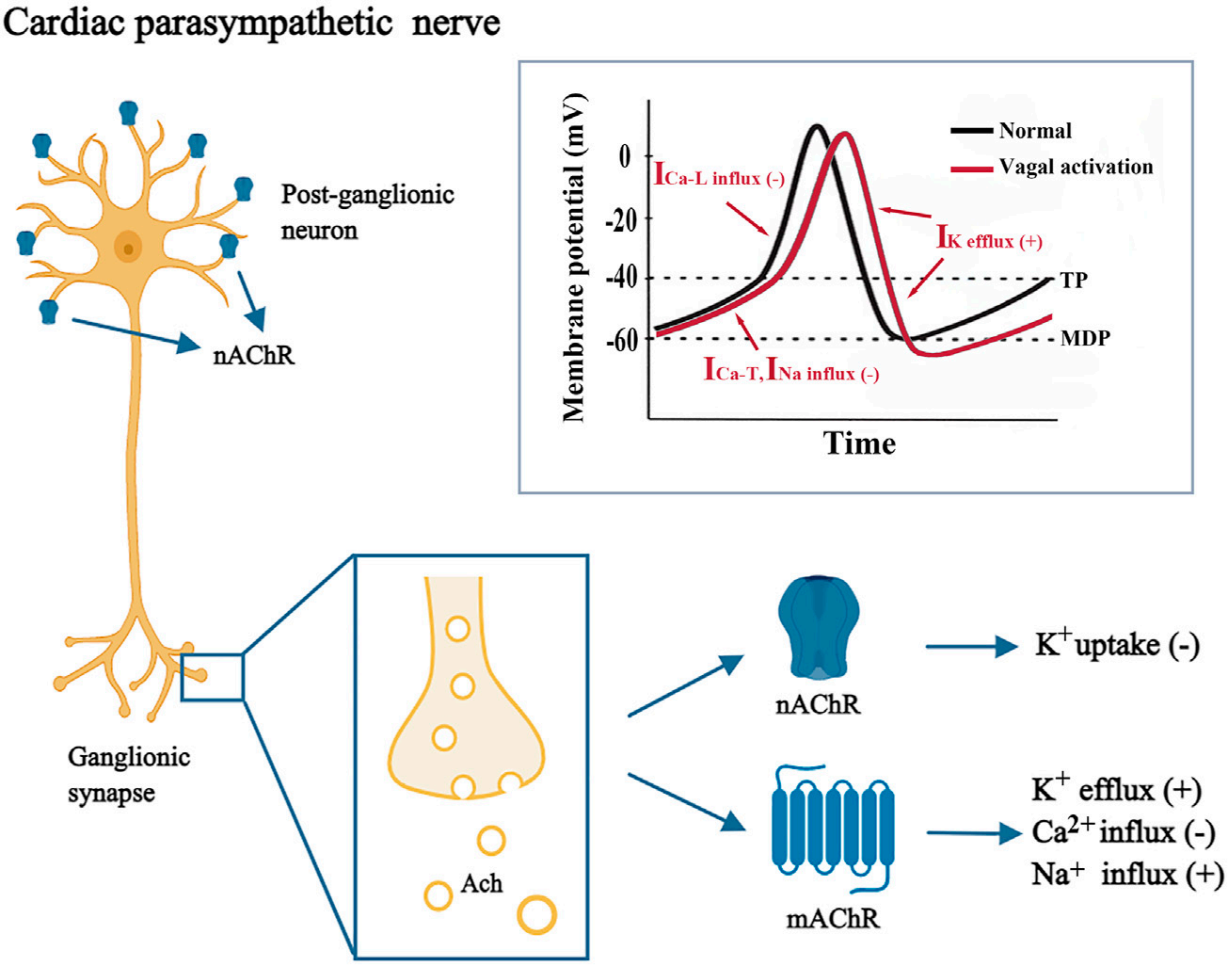
Mechanism underlying vagal-induced sinus node dysfunction. The gray box shows the waveform of sinoatrial node action potentials under normal condition and during vagal activation. ACh, acetylcholine; I_Ca-L_, L-type calcium current; I_Ca-T_, T-type calcium current; I_Na_, sodium current; I_K_, potassium current; Ca^2+^, calcium ion; K^+^, potassium ion; MDP, maximum negative diastolic potential; mAchR, muscarinic acetylcholine receptor; Na^+^, sodium ion; nAChR, nicotinic acetylcholine receptor; TP, threshold potential.

The mechanism underlying SND development is not fully understood; however, evidence suggests that it may be caused by changes in the sinus node itself (degenerative diseases, myocardial ischemia or infarction, inflammation, and endocrine abnormalities) or extrinsic factors such as autonomic imbalance (Kusumoto et al., 2019). According to the European Society of Cardiology (ESC) guidelines on cardiac pacing, hypervagotonia is a potentially reversible or treatable cause of SND (Glikson et al., 2021). The neurophysiological mechanisms underlying vagal overactivity include the recruitment of vagal neurons, more efficient transmission at the level of the ganglia, and/or just increased excitability of vagal neurons, although this apparent plasticity of the nervous system remains unknown. The basic pathophysiological mechanism of functional SND (Figure 1) is that the ACh released from excited vagal nerve endings, which bind to M2 muscarinic ACh receptors (mAChRs) on the cell membrane, increase potassium permeability and facilitate the efflux of potassium ions (K+). Besides that, the inhibition of K^+^ uptake may occur through stimulation of the nicotinic ACh receptors (nAChRs) on the sinus node cells (Bibevski and Dunlap, 2004). Owing to hyperpolarization of the sinus node cell membrane, and the inward current of the phase 4 depolarization of the action potential is inhibited, the automatic depolarization rate decreases, ultimately resulting in changes in auto-rhythmicity and chronotropism of the sinus node (Doyen et al., 2019). There is evidence that, nAChRs mediate a large part of ganglionic transmission in canine intracardiac neurons, suggesting that active nAChRs may promote the activity of the vagus nerve (Bibevski et al., 2000). In hence, the M2 mAChRs mainly mediate cardiac myocyte signaling, whereas nAChRs maybe mediate both signaling at the postganglionic neuron and cardiac myocyte.

An animal study has demonstrated that surgical dissection of the fat pad overlying the junction between the right pulmonary and left atrial veins can interrupt the vagal inputs to the sinoatrial node region (Randall et al., 1987). In humans, a preliminary attempt at selected ablation of the parasympathetic nerves surrounding the sinus node (cardiac ganglia would not be damaged) for treating bradyarrhythmia patients achieved good results (Lu et al., 2020). Also, we have previously reported specific parasympathetic plexus fibers that significantly affected the heart rate (Hu et al., 2019). Therefore, theoretically, in functional SND patients who select the treatment, modification of cardiac vagal ganglia would help to improve the symptoms resulting from bradyarrhythmias.

## 4 New treatment for refractory symptomatic sinus node dysfunction: Cardioneuroablation

In the current guidelines, symptomatic SND associated with bradyarrhythmias is an indication for permanent pacing therapy (Class of Recommendation I, Level of Evidence B) (Glikson et al., 2021). To date, there has been no clear recommendations proposed for symptomatic sinus bradycardia or sinus pauses that are secondary to elevated vagal tone (Kusumoto et al., 2019; Glikson et al., 2021). For younger patients with major symptoms, without organic sinus node damage, and ineffective drug therapy, choosing cardiac pacing as a therapeutic strategy would be concerning due to long-term effects.

Cardiac autonomic modification by an interventional method with minimal invasion is a new treatment idea; most previous curative effective studies have focused on VVS patients (Pachon et al., 2005; Pachon et al., 2011; Yao et al., 2012; Aksu et al., 2016; Debruyne et al., 2018; Hu et al., 2019; Aksu et al., 2020; Pachon et al., 2020; Aksu et al., 2021; Debruyne et al., 2021; Tu et al., 2022) and shown high efficacy and safety. GP ablation can restore the balance between sympathetic and parasympathetic components and change their effects on diverse physiological functions of the heart. Thus, the modulatory effect of CNA on the vagal nerves indicates CNA may also be effective in patients with functional SND. Previous studies involving patients with SND who underwent CNA comprised several mixed cohort studies that included patients with VVS, SND, or FAVB (Table 1) (Pachon et al., 2005; Pachon et al., 2011; Yao et al., 2012; Zhao et al., 2015; Aksu et al., 2016; Qin et al., 2017; Debruyne et al., 2018; Hu et al., 2019; Aksu et al., 2020; Pachon et al., 2020; Aksu et al., 2021; Debruyne et al., 2021; Tu et al., 2022). Application of CNA to overcome the deleterious effects of enhanced vagal tone in bradyarrhythmia-related cases was originally reported by Pachon et al. (Pachon et al., 2005). None of the patients with SND had syncope recurrence in the 9.2 ± 4 months following GP ablation. Subsequently, two single cohort studies have investigated the efficacy of CNA for treating symptomatic sinus bradycardia in non-elderly patients (Zhao et al., 2015; Qin et al., 2017). In one study, during a follow-up of 18.4 ± 6 months, all patients reported significant symptom improvement (Zhao et al., 2015); similar results were repeated by Qin et al. (Qin et al., 2017). With partial ablation of the cardiac ganglionated plexus, Debruyne et al. demonstrated good efficacy by using more restricted selection criteria before the procedure and through a computed tomographic-guided procedure in patients with SND (Debruyne et al., 2018).

**TABLE 1:** Studies on cardioneuroablation for sinus node dysfunction.

Authors	*N*	Age (years)	GP localization method	Pre-ablation burden^d^	Post-ablation burden^d^	Pre- cSNRT (ms)	Post-cSNRT (ms)	Other pre-ablation functional data^e^	Other post-ablation functional data^e^	Mean follow-up (months)
Pachon 2005 Pachon et al. (2005)	13^a^	47.5 ± 16^b^	SA + AA	77%	8%	578.9 ± 288	261.9 ± 97	54 ± 7 bpm	71 ± 10 bpm	9.2 ± 4
Zhao 2015 Zhao et al. (2015)	11	45.9 ± 11	HFS + AA	10.7 ± 2	3.7 ± 2	400.8 ± 122	302.1 ± 162	51 ± 4 bpm	61.7 ± 5 bpm	18.4 ± 6
Aksu 2016 Aksu et al. (2016)	7^a^	42.1 ± 15	SA + HFS + AA	10.8 ± 3	1.1 ± 1	612 ± 55	394 ± 24	53.8 ± 17 bpm	74.3 ± 11 bpm	9.5 ± 3
Qin 2017 Qin et al. (2017)	62	47.8 ± 13	AA	100%	8%	423.9 ± 105	NS	47.3 ± 6 bpm	63.5 ± 8 bpm	12
Debruyne 2018 Debruyne et al. (2018)	8^a^	47.5 ± 21	AA^c^	24	1	232 ± 176^f^	132 ± 107^f^	983 ± 108 ms	764 ± 169 ms	6
Debruyne 2021 Debruyne et al. (2021)	19^a^	46.9 ± 19	AA^c^	72	3.6	425 ± 577	232 ± 170	998 ± 172 ms	782 ± 147 ms	12

^a^ Number of patients with sinus node dysfunction in the mixed-case cohort.

^b^ Mean age of all patients in the mixed-case cohort.

^c^ Electroanatomical map merged with the computed tomographic image.

^d^ Percentage of symptomatic in (Chiou et al., 1997); symptom score in (Yao et al., 2012); number of prodromal symptoms in (Hu et al., 2019); percentage of patients with obvious or recurrent symptoms in (Pachon et al., 2020); syncope burden in (Tu et al., 2022) and (Aksu et al., 2016).

^e^ Functional data refer to the common indicators for observing the heart rate in CNA-related studies (mean heart rate in (Chiou et al., 1997; Yao et al., 2012; Hu et al., 2019; Pachon et al., 2020); P-P interval in (Tu et al., 2022) and (Aksu et al., 2016).

^f^ Total corrected sinus node recovery time in the mixed-case cohort. AA, anatomical approach; cSNRT, corrected sinus node recovery time; GP, ganglionated plexus; HFS, high-frequency stimulation; N, number; NS, not stated; SA, spectral analysis.

**FIGURE 2:**
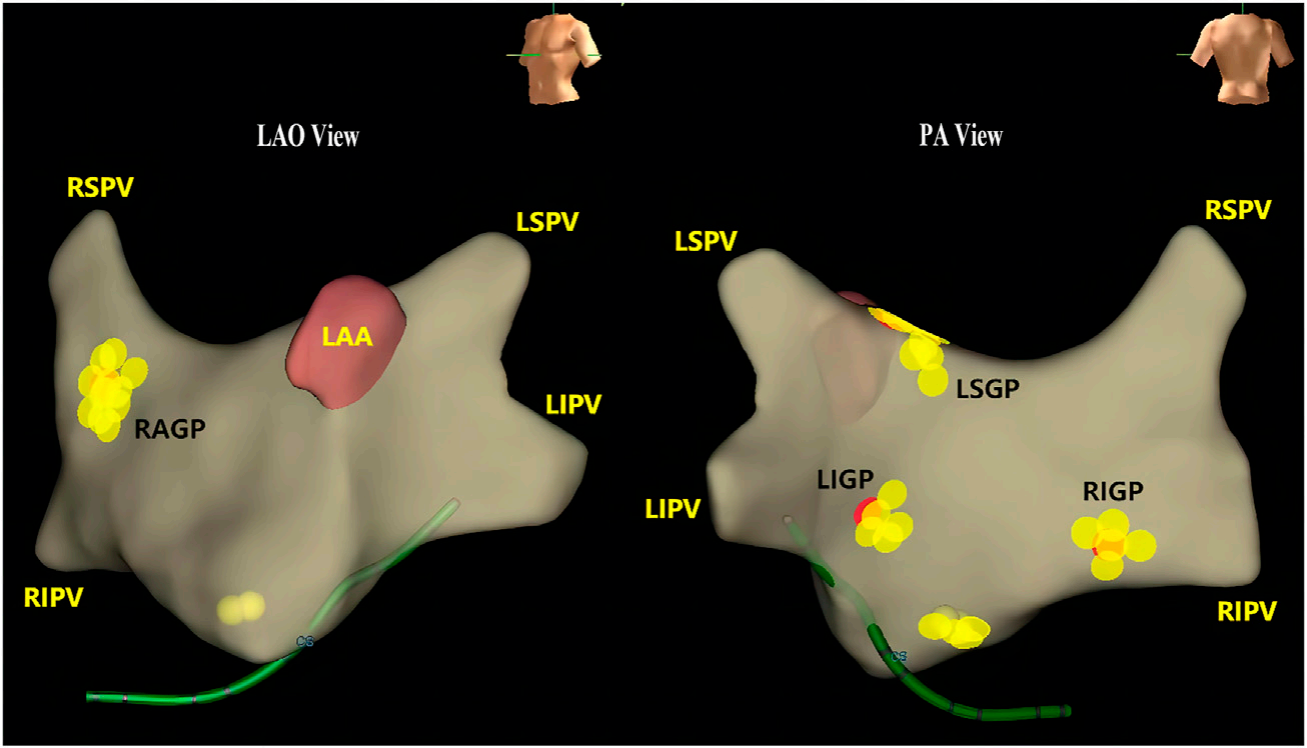
Location of the cardiac ganglionic plexus. Three-dimensional endocardial surface of the left atrium and locations of the ganglionated plexi (GPs) of a patient with vasovagal syncope. Yellow points indicate the locations of the GPs. Red points indicate the ablation sites. LAA, left atrial appendage; LIGP, left inferior ganglionated plexus; LIPV, left inferior pulmonary vein; LSGP, left superior ganglionated plexus; LSPV, left superior pulmonary vein; RAGP, right anterior ganglionated plexus; RIGP, right inferior ganglionated plexus; RIPV, right inferior pulmonary vein; RSPV, right superior pulmonary vein.

Four cardiac vagal GPs on the surface of the atrium are the major ablation sites (Manolis et al., 2021), generally located at junctions of the left atrium and four pulmonary veins: left superior GP (LSGP), left inferior GP (LIGP), right anterior GP (RAGP), and right inferior GP (RIGP) (Figure 2). It has been reported that sinus nodes are mainly innervated by nerve fibers from the RAGP, and electrically stimulating the RAGP can decrease the sinus rate and shorten the effective refractory period of peripheral atrial cells but does not affect atrioventricular nodal conduction (Hou et al., 2007). Consistent with this study, we have previously reported that ablation of the RAGP could immediately increase heart rate and maintain stability for a long period (Hu et al., 2019). The other target GP that can significantly increase heart rate is located within the fat pad between the superior vena cava and the aortic root (SVC-Ao); the SVC-Ao fat pad is the “head station”, which connects the extrinsic with the intrinsic cardiac autonomic nervous system (Qin et al., 2017). Most efferent cardiac vagal fibers to the atrium, sinus node, and atrioventricular node pass through this area and then project onto the RAGP (Armour et al., 1997; Chiou et al., 1997). A recent study confirmed that the ablation of SVC-Ao GP significantly increased the heart rate, while elimination of vagal response evoked by extracardiac vagal stimulation (Chen et al., 2022). The RAGP and SVC-Ao GP were considered more valuable candidate targets to modulate sinus node function in symptomatic SND patients when compared with other GPs. Notably, the ablation of SVC-Ao GP may change the electrophysiological function of the heart, such as prolonging the effective refractory period acutely, while shortening regional ERPs, which increase atrial fibrillation or tachycardia burden chronically (Lo et al., 2013). Exploration of the anatomical principles of the innervation of the heart is ongoing, which will improve the scheme for modern therapies to directly target cardiac autonomic nervous function.

CNA remains an emerging modality that did not eliminate vagal innervation of the whole heart, rather modified the degree of innervation of the overactive vagus nerve fibers, especially in SND patients conducted sinus node-related regional GP ablation protocols. Although increased heart rate is consistent with the expected effect of CNA, inappropriate sinus tachycardia is a common short-term adverse event in VVS patients (Vandenberk et al., 2022). However, only a minority of SND patients experience palpitations without recording arrhythmia events, which may be related to the patient’s short-term maladjustment to the significantly increased heart rate (Zhao et al., 2015; Qin et al., 2017).

## 5 Selection criteria for patients undergoing cardioneuroablation based on supporting evidence

### 5.1 Preliminary workup

Evaluation of patients with suspected SND requires a combination of history, physical examination, electrocardiography (ECG), and imaging to define symptom-related bradyarrhythmia (Kusumoto et al., 2019). Patient history should be limited to bradycardia-derived symptoms, documenting their frequency, severity and duration. The relationship between symptoms and emotional or psychological distress, physical activity, positional changes, medical measures, and typical triggers (cough, prolonged upright posture, urination, and defecation) should be explored along with pulse rate change, if measured during an episode (Kusumoto et al., 2019). In addition, family history provides data on irreversible genetic abnormalities, including familial SND, inherited cardiomyopathies, or inheritable arrhythmic conditions associated with SND (catecholaminergic polymorphic ventricular tachycardia and long QT syndrome) that are often attributed to gene mutation (Manoj et al., 2022). Physical examination and a detailed history help exclude systemic diseases, drug effects, and surgical trauma. ECG is used to confirm the rate and rhythm, the nature and degree of conduction disorder, and to record specific performance of cardiac electrical activity that indicates possible abnormalities such as systemic or structural heart disease (high and low QRS voltage, prolonged corrected QT interval, pathological Q waves). Twenty-four-hour Holter monitoring can refine diagnosis or determine a symptom-rhythm correlation; CNA requirement threshold is a <50 beats per minute heart rate that arrests more than 3 s at symptom onset. Laboratory tests (serum potassium or thyroid function) are reasonable if underlying causes are suspected; echocardiography and cardiac magnetic resonance imaging can reveal abnormal cardiac structures directly. Identifying abnormalities at the molecular level often requires genetic testing. For patients with daily symptoms, either ECG or 24-h continuous ambulatory ECG is more suitable. For patients with unexplained or intermittent symptoms (>30 days between symptoms), individualized long-term monitoring with mobile cardiac outpatient telemetry or implantable cardiac monitoring may help capture abnormal heart rhythm during symptom onset (Kusumoto et al., 2019). If syncope is the primary manifestation with no arrhythmia, cardioinhibitory VVS must be ruled out. VVS is commonly triggered by maintaining an upright posture for an extended period or by stressors (emotional stress, pain, or exercise), features autonomic reflex responses (diaphoresis, warmth, nausea, and pallor), and is followed by fatigue; syncope characteristics and a tilt test can assist diagnosis (Writing Committee Members et al., 2017).

The basic condition for screening SND candidates is a clarification of non-organic pathological changes by the assessment processes described above.

### 5.2 Qualitative assessment of sinus node function

After initial assessment, testing associated with sinus node function is what follows, such as atropine test (AT), electrophysiological study, and exercise test. A definitive protocol for the AT among current guidelines is lacking, but this test is chosen to serve as a screening tool for CNA candidates in all studies involving SND (Pachon et al., 2005; Zhao et al., 2015; Aksu et al., 2016; Qin et al., 2017; Debruyne et al., 2018; Aksu et al., 2020; Aksu et al., 2021; Debruyne et al., 2021). The application of other examinations has not been unified.

#### 5.2.1 Atropine test

Observing the heart rate response through pharmacologic interventions is not frequently warranted in clinical practice, but the evaluation of the intrinsic heart rate might be helpful if there is a question regarding intrinsic versus extrinsic SND. As an anticholinergic agent, atropine can simulate the effect of vagal denervation, manifesting an acceleration of both spontaneous depolarization of sinoatrial node pacemaker cells and atrioventricular conduction, which increases heart rate (Manoj et al., 2022). Thus, dysfunction of the sinus node in a patient with bradyarrhythmia, which might be attributed to vagal overactivity, could restore partly or completely when vagal neural control was largely eliminated using atropine. In this sense, it is worthwhile considering CNA for the treatment of patients who have indications of pacemaker implantation but without structural cardiopathy.

A positive AT result may suggest the possibility of intrinsic SND, while there is no unified standard for specific protocols and results. There were five different statements regarding the AT protocol and positive criteria (Table 2). The possibility of irreversible lesions of the sinus node should be considered if there is no or slight response to atropine; however, these outcomes cannot completely rule out the vagal inhibitory effect. The following conditions may contribute to a mismatch between the AT outcomes and the actual situation: 1) using a conventional dose of atropine in overweight or obese subjects is not sufficient to provoke noticeable response owing to the limitation in maximum dosage; low-dose atropine may in cause paradoxical bradycardia (Mandel et al., 1972); 2) subjects are insensitive to atropine; 3) Differences in the amount and quality of the cholinergic receptors among individuals (Vavetsi et al., 2008); 4) taking drugs that would suppress sinus node pacemaking; 5) high sympathetic tone partially masks the intrinsic sinus node involvement.

**TABLE 2:** Atropine test protocols in different studies.

Authors	N	Age (years)	Dose	ECG recording	Positive result^b^
Zhao 2015 Zhao et al. (2015)	11	45.9 ± 11	0.03 mg/kg	NS	HR < 90 bpm within 20 min or find out junctional rhythm, sinus bradycardia, or sinus pause
Aksu 2016 Aksu et al. (2016)	7	42.7 ± 15^a^	0.04 mg/kg	continuous recording for 30 min	HR increase of <25% within 15 min
Qin 2017 Qin et al. (2017)	62	47.8 ± 13	0.03 mg/kg	NS	HR <90 bpm within 20 min, or find out junctional rhythm or sinus pause
Debruyne 2018 Debruyne et al. (2018)	8	47.5 ± 21	2 mg (two injections, 1 mg per time)	6 sequential ECG recordings of >1 min: 4 before atropine injection, 1 > 3 min after injection of 1 mg atropine, and the last >3 min after a second bolus of 1 mg (if no significant response after first injection). The whole procedure typically took 30 min	P-P interval shortening <20% and ≥1,000 ms
Aksu 2020 Aksu et al. (2020)	15	39.6 ± 14^a^	0.04 mg/kg	continuous recording for 30 min	HR increase of <25% or HR < 90 bpm within 20 min

^a^ Mean age of all patients in the mixed-case cohort.

^b^ Positive results indicate organic sinus node lesions. ECG, electrocardiogram; HR, heart rate; N, number; NS, not stated.

In a study that analyzed autonomic influences, comparison of the electrophysiological variables between two autonomic states and a basal state in individuals <60 years old showed a significant difference in those under isolated parasympathetic blockade compared with those under full autonomic blockade (de Marneffe et al., 1986). Therefore, selection of functional SND patients *via* AT is more reliable than combined autonomic blockade. When patients have contraindications of atropine, such as prostatomegaly and glaucoma, diagnostic electrophysiological assessment should be conducted.

#### 5.2.2 Electrophysiological study

The purpose of an electrophysiological study, in the context of bradycardia assessment, is to distinguish Intrinsic SND and disturbed autonomic regulation. Sinus node recovery time (SNRT) is the most straightforward indicator of sinus node function, which is making use of the physiological characteristics of sinus node pacemaking, namely “overdrive suppression,” to measure the interval between the last driven atrial depolarization and the next spontaneous atrial depolarization (Pick et al., 1951).

A commonly used form of SNRT is the corrected SNRT (cSNRT), which is obtained by subtracting the baseline sinus cycle length from the SNRT; the value of cSNRT in normal adult ranges from 500 to 550 ms (most commonly <525 ms) (Sathnur et al., 2021). Some reports have demonstrated excellent success in the group with abnormal cSNRT (Aksu et al., 2020; Aksu et al., 2021), although cSNRT over 525 ms (considered a positive indication of intrinsic changes of the sinus node) was used as an exclusion criterion in two purely SND studies (Zhao et al., 2015; Qin et al., 2017). Due to the invasiveness of electrophysiological studies, cSNRT is more often used as a reference for treatment outcome or support for diagnosis before the procedure (Table 1). Another indicator is sinoatrial conduction time (SACT), but its application value is inferior to cSNRT, and it is rarely used in clinical practice.

Electrophysiologic evaluation of sinus node function by utilizing combined autonomic blockade is common in the pathophysiological study of SND. To evaluate the intrinsic sinus node function separated from autonomic control, intrinsic heart rate (IHR), intrinsic cSNRT, and intrinsic SART are measured following administration of atropine and propanolol (Szatmáry et al., 1983; de Marneffe et al., 1986; Marcus et al., 1991). IHR reflects the capacity of spontaneous depolarization of the sinus node without autonomic effect, therefore, only disturbed autonomic regulation was regarded as the responsible mechanism of SND in patient with normal IHR (Marcus et al., 1991). Yet different parameter values were reported, possibly attributed to the non-standardized methodology and the variable patient characteristics. A negative electrophysiological study cannot exclude an intrinsic lesion in the sinus node but could be a secondary verification of sinus node function based on negative AT, since the etiology of SND can be established preceded by invasive evaluation in most cases.

#### 5.2.3 Exercise test

Exercise test has a limited role in SND: its main application is to confirm a suspected diagnosis of chronotropic incompetence (Evrengul et al., 2006). The combination of vagal withdrawal and sympathetic activation can lead to an accelerated heart rate during exercise, and a decrease in heart rate (heart rate recovery) immediately following exercise is deemed to be an indicator of parasympathetic activity (Evrengul et al., 2006). An exercise test with adequate sinus chronotropic response could provide evidence of functional SND (Aksu et al., 2016). Bruce treadmill protocol allows for recording heart rate at different phases between rest and peak exercise and improving the comprehensive evaluation of chronotropic and autonomic functions (Evrengul et al., 2006). Our target patients are relatively young and healthy, hence are the optimal subjects for the Bruce protocol (Evrengul et al., 2006). Failure to reach 80% of the heart rate reserve [the difference between the maximal predicted heart rate (220 minus age) and the resting heart rate] during exercise was equivalent to an impaired chronotropic response (Evrengul et al., 2006). However, some patients with SND could reach target heart rates comparable to that of the normal control if forced to exercise, even though the time taken to reach such heart rates may be longer, with increase physical exertion.

Old age, comorbidities, and drug-related exercise intolerance may have an impact on the results of exercise tests, but these factors are usually excluded since these patients are relatively young and healthy.

### 5.3 Quantitative measurements of vagal activity

A significant step in deciding whether a patient is suitable for CNA is to determine the extent of contribution of hypervagotonia to the occurrence of clinical symptoms, but the quantitative evidence of vagal overactivity in previous SND-related studies is lacking.

Minor time variation between adjacent heartbeats indicates that the sympathetic and vagal nerves modulate heart rate through the regulation of sinus nodes in parallel or independently. On this basis, heart rate variability (HRV) has been identified as a non-invasive indicator that can reflect the autonomic control of the heart and can be drawn from 12-lead ECG or ambulatory ECG monitoring (Sassi et al., 2015). The common types of analyses used to evaluate HRV include time-domain and frequency-domain analyses (Sassi et al., 2015). Time-domain indices, such as the standard deviation of all N-N intervals (SDNN) and HRV triangular index, can be obtained by statistical and geometrical measures, respectively (Sassi et al., 2015). Frequency-domain indices are derived by decomposing the varying R-R interval or instantaneous heart rate into multiple frequency-domain components of different powers, such as low frequency (LF) and high frequency (HF) (Sassi et al., 2015). The current consensus is that root mean square of successive differences between normal heartbeats (RMSSD), percentage of successive N-N interval differences that are over 50 milliseconds (pNN50), and HF are the most common markers of vagal activity. An animal study has shown that common indices of HRV are not correlated with the levels of vagal tone, which were directly measured in rats with and without anesthesia (Marmerstein et al., 2021). The author considered respiratory sinus arrhythmia as the cause of vagal components of HRV. The “non-linear” relationship between neural activity and sinus cycle length has been illustrated in a previous study; the phenomena may result in an intrinsic rate-dependency of autonomic indices, but normalized frequency-domain indices (the LF/HF ratio) appear to be devoid of intrinsic rate-dependency (Rocchetti et al., 2000; Zaza and Lombardi, 2001). HRV is an indirect measure of vagal activity that is susceptible to interference from several factors (respiration, physical exercise, and emotional stress) (Malik et al., 2019; Marmerstein et al., 2021); however, as a preliminary assessment tool for autonomic nervous tone, it is still applicable if these interfering factors are controlled.

Only independent changes not related to fluctuation in the mean heart rate are strongly suggestive of an improved cardiac vagal output, while time-domain HRV analysis cannot extract the key information within the mean heart rate variation due to the associated complex physiological changes. This shortcoming of HRV is compensated by a newly proposed metric. Cardiac deceleration capacity (DC) was reported as a predictive marker of mortality after myocardial infarction in 2006 and can be obtained by processing the sequences of R-R intervals with the phase-rectified signal averaging (PRSA) technique after Holter recordings: 1) setting the R-R intervals to longer than the former interval as anchors, meanwhile to avoid errors caused by artifacts, intervals that prolonged >5% are removed; 2) segments of equal length around the anchors are selected and aligned at the anchors; 3) signals X(i) within the aligned segments are averaged to obtain the data required for the next steps, X(0) and X(1) are calculated *via* averaging the R-R intervals of the anchor points and its following R-R intervals, X(−1) and X(−2) represents the average of the two R-R intervals preceding the anchor points; 4) Using the equation: DC = [X(0)+X(1)−X(−1)−X(−2)]/4, DC can be quantified and calculated for the entire 24 h, or calculated separately for two periods of the day [from 8:00 to 23:00 (daytime DC) and 23:00 to 8:00 (nighttime DC)] (Bauer et al., 2006a). Increased cardiac vagal activity could slow down the heart rate and enhance the deceleration capacity. Vagal activity can be quantified by measuring DC, and the reduction in DC reflects a decrease in vagal regulation of the heart (Bauer et al., 2006b; Sheldon et al., 2015). We have previously analyzed the clinical value in VVS patients and found that DC > 7.5 ms may indicate abnormally heightened cardiac vagal tone and could be used for the diagnosis of VVS (Zheng et al., 2020). DC has shown superior predictive value and extra clinical benefit in VVS patients as a part of an innovative enrollment strategy that not only elevates the success rate of ablation but also benefits patients who were once thought ineffective with CNA (Tu et al., 2022). Compared with HRV, DC can extract periodic components of the vagal nerve modulation process; non-periodic interfering components, such as noisy artifacts or arrhythmias, can be removed using the PRSA algorithm (Bauer et al., 2006a). Thus, detection of DC may be a new method to quantify cardiac vagal tone and could play a role in defining indications for CNA.

Interestingly, a previous clinical study has reported that manifestations of vagal response in patients with symptomatic sinus bradycardia during ablation were in stark contrast to VVS: the former showed heart rate drops or junctional rhythm (but no patients presented a sinus pause over 2 s) while the latter showed sinus pause over 3 s (Zhao et al., 2015). These previous findings suggest that the underlying differences in vagal inhibition degree between symptomatic sinus bradycardia and VVS deserve further exploration.

### 5.4 Age-related sinus node dysfunction

Symptomatic sinus bradycardia and sinus pauses caused by autonomic imbalance usually occur in younger patients without significant structural cardiac disease. The progressive disappearance of nodal cells or the pathological infiltration of fibrous tissue within sinus node with advanced age is more likely to become the etiology of the dysfunctional sinus node (Csepe et al., 2015). Intrinsic SND occurs most frequently in the elderly, especially those aged ≥70 years, in which most occurrences are due to age-related damage in the sinus node, and associated with heart failure, sinus node ischemia, and inflammatory conditions (Jensen et al., 2014; Csepe et al., 2015). Indeed, natural increase in sinus node fibrosis is necessary to optimize electrical insulation from the surrounding atrial myocardium and safe propagation and is not directly associated with SND. However, the degenerative loss of pacemaker cells and progressive fibrosis are evident as shown from specimens isolated from SND patients (Csepe et al., 2015). When fibrosis is upregulated in cardiac diseases, the role becomes pathophysiological.

Evidence has suggested that the vagal excitation extent has age-related variation; indeed, responses of younger patients to atropine are more significant than those of older patients, with a higher number of evoking vagal responses during the procedure (Zhao et al., 2015). This age correlation has also been suggested by de Marneffe et al. who reported that the autonomic control in young individuals (<60 years of age) is characterized by the predominance of vagal activity, while in older individuals (≥60 years of age) with or without sinus node disease, sympathetic activity is more prominent (de Marneffe et al., 1986). In parallel with aging of the sinus node, the unchanged sympathetic activity and progressive decrease in vagal activity contribute to the basal properties of sinus node remaining stable throughout life (de Marneffe et al., 1986; de Marneffe et al., 1993). Therefore, old individuals with functional SND may have exaggerated vagal tone without sufficient compensation by age-related relative superiority of the sympathetic role. The significant increase in heart rate following atropine treatment cannot exclude the contribution of the relatively dominant sympathetic nerve.

Two studies have discussed the influence of age on CNA effect in symptomatic SND patients. In the first study (Qin et al., 2017), the patients were pre-specified into group A (<50 years of age) and group B (≥50 years of age). The authors found that patients undergoing anatomically guided atrial autonomic ablation yielded better clinical outcomes, including increased sinus rate and improved quality of life, in group A than B. The recovery speed of autonomic balance in the younger group was also faster than that in the older group from 3 days to 12 months post-CNA. Conversely, in the second study (Debruyne et al., 2021) of three patients with syncope recurrence and corroboration of a sinus pause, two were ≥70 years and at a greater risk of intrinsic sinus node injury, and finally underwent pacemaker implantation after 6 months. Of note, they were in the early stages of intrinsic SND as their heart rate increased moderately following pre-ablation AT.

In summary, SND patients recommended for CNA should be under 60 years of age. Except for the insufficient therapeutic effect in old patients, the cardioprotective role of vagal activity is another important consideration (Bauer et al., 2006a), therefore, pacemaker implantation to maintain heart rate would be a more reasonable option, instead of increasing the cardiac work and myocardial electrical instability in elderly patients.

## 6 Thinking about the future

**FIGURE 3:**
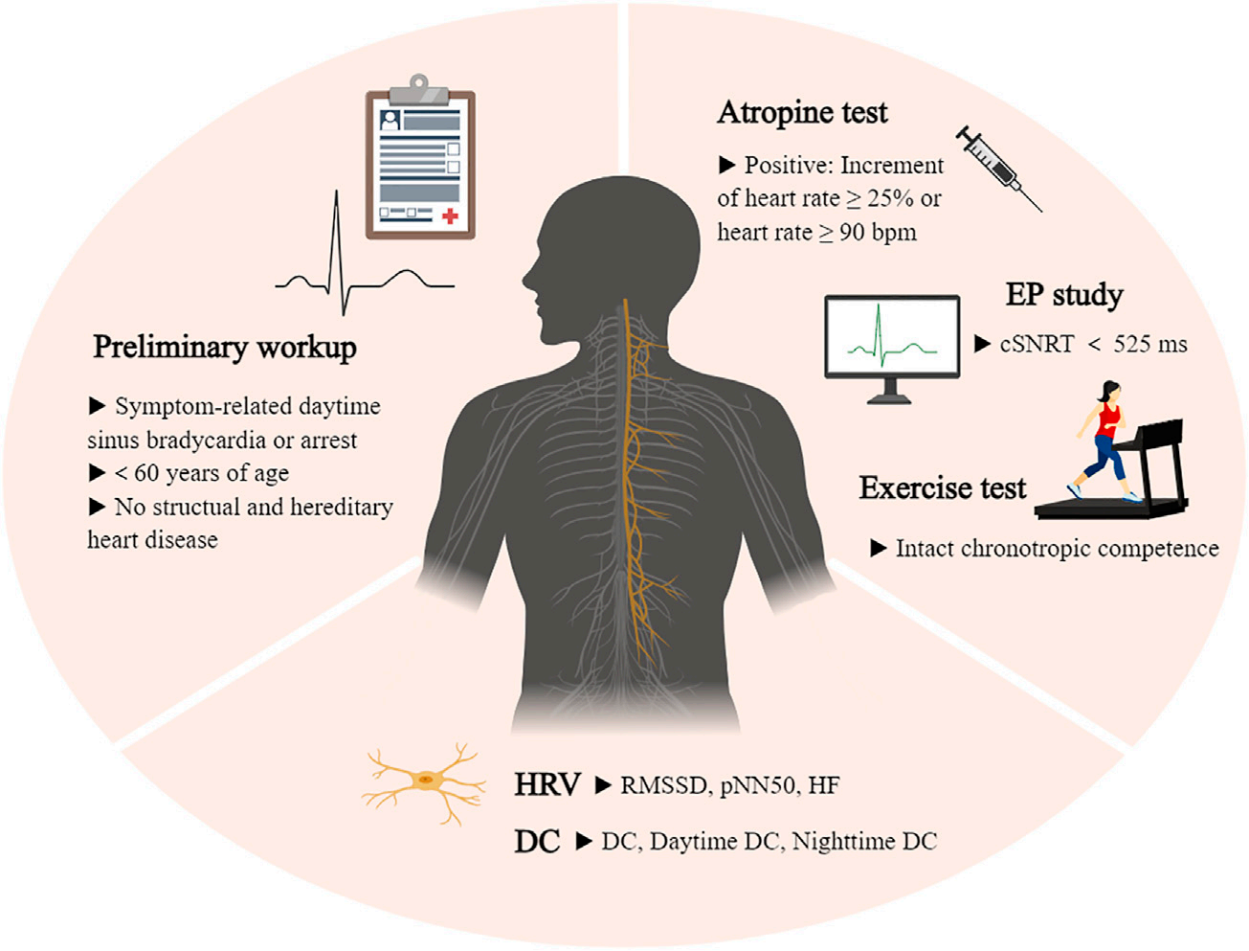
Illustration of the criteria process. Selection criteria for patients with sinus node dysfunction undergoing cardioneuroablation. cSNRT, sinus node recovery time; DC, deceleration capacity; EP study, electrophysiological study; HF, high frequency; HRV, heart rate variability; pNN50, percentage of successive N-N interval differences over 50 ms; RMSSD, successive cardiac cycle difference value mean square root.

Based on the above evidence, the following selection criteria will help to decide potential candidates: symptom-related daytime sinus bradycardia (<50 beats per minute) or arrest (>3 s); age <60 years; no structural and hereditary heart disease; positive AT (increment of heart rate ≥25% or heart rate ≥90 beats per minute) and/or abnormal electrophysiological study; intact chronotropic competence in exercise test; raised vagal activity (Figure 3).

Due to the absence of a gold standard for diagnosing functional SND and a lack of large sample size studies, the sets of criteria described above are based on previous reports and still need further improvement. Screening indicators have pros and cons and should therefore be selected and combined according to the specific situation (Table 3). In exclusive functional SND, electrophysiological means should only be used as an exclusionary diagnostic tool. But even with rapid atrial stimulation and autonomic blockade means, negative results can be observed in intrinsic SND. HRV can be applied to initially assess the overall balance of the autonomic nervous system, and further measurement of vagal tone using DC analysis is a better option. Examinations that reflect vagal activity should be supplemented in future research. Notably, the diagnostic value of DC has been proven only in patients with VVS aged approximately 40 years (Zheng et al., 2020).

**TABLE 3:** Comparison of assessment methods for sinus node function and vagal activity.

Methods	Basic principle	Observation indices	Advantages	Disadvantages
Qualitative assessment of sinus node function				
Atropine test	Using muscarinic cholinergic receptor antagonist (atropine) to simulate the effect of vagal denervation	Heart rate and its increment	Easy operation and wide clinical application	Adverse drug reactions (mouth dryness and palpitation); atropine contraindications (prostatomegaly and glaucoma)
Electrophysiological study	Overdrive pacing suppresses sinus node automaticity; sinus cycle recovers after the last driven	Baseline: Resting HR, cSNRT Autonomic blockade: intrinsic HR; intrinsic cSNRT	Provides accurate information on the electrophysiological function of the sinus node	Invasive method; no clear critical value for the indices after nerve blockade
Exercise test	Autonomic regulation of HR during and after exercise	Maximum HR; Heart rate recovery	Definitive diagnosis of chronotropic incompetence and evaluation of autonomic function	Exercise intolerance related to factors other than abnormality in the intrinsic dysfunction of the sinus node has an impact on the results
Quantitative assessment of vagal activity				
Heart rate variability (HRV)	HRV represents the capacity of HR response for dynamic changes while under autonomic regulation, which is manifested as the minor time variation between adjacent heartbeats; DC derived from HRV, extracts only vagal-related deceleration cycle and removes non-periodic interfering components *via* the PRSA algorithm	RMSSD, pNN50, HF	The value of its application has been proven by extensive data	Susceptible to external factors (respiration, physical exercise, and emotional stress)
Deceleration capacity (DC)		DC, Daytime DC, Nighttime DC	Directly measured the vagal tone; no explicit relevance with baroreflex, respiratory, or central autonomic influence	The value of its application in SND patients is unknown

cSNRT, sinus node recovery time; HF, high frequency; HR, heart rate; pNN50, percentage of successive N-N interval differences over 50 ms; PRSA, phase-rectified signal averaging; RMSSD, successive cardiac cycle difference value mean square root; SND, sinus node dysfunction.

## 7 Conclusion

As a feasible therapy, the moderation effect of vagal nerve by CNA increases heart rate and eliminates bradyarrhythmia-related symptoms in younger patients with functional SND. In this review, we summarized a set of selection criteria for the identification of the best candidates to enhance the success rate of CNA. However, the diagnostic accuracy of the truly “vagally mediated SND” is challenging because most of the studies have small sample size and lack quantification of vagal overactivity, resulting in evidence of limited value. Future comprehensive, large sample studies are warranted to remedy this situation.

## References

[B1] Aksu T. Golcuk E. Yalin K. Guler T. E. Erden I. (2016). Simplified cardioneuroablation in the treatment of reflex syncope, functional AV block, and sinus node dysfunction. Pacing Clin. Electrophysiol. 39 (1), 42–53. 10.1111/pace.12756 26411271

[B2] Aksu T. Guler T. E. Bozyel S. Golcuk S. E. Yalin K. Lakkireddy D. (2021). Medium-term results of cardioneuroablation for clinical bradyarrhythmias and vasovagal syncope: Effects on QT interval and heart rate. J. Interv. Card. Electrophysiol. 60 (1), 57–68. 10.1007/s10840-020-00704-2 32034611

[B3] Aksu T. Guler T. E. Bozyel S. Yalin K. (2020). Vagal responses during cardioneuroablation on different ganglionated plexi: Is there any role of ablation strategy? Int. J. Cardiol. 304, 50–55. 10.1016/j.ijcard.2019.12.003 31836362

[B4] Armour J. A. Murphy D. A. Yuan B. X. Macdonald S. Hopkins D. A. (1997). Gross and microscopic anatomy of the human intrinsic cardiac nervous system. Anat. Rec. 247 (2), 289–298. 10.1002/(SICI)1097-0185(199702)247:2<289:AID-AR15>3.0.CO;2-L 9026008

[B5] Bauer A. Deisenhofer I. Schneider R. Zrenner B. Barthel P. Karch M. (2006). Effects of circumferential or segmental pulmonary vein ablation for paroxysmal atrial fibrillation on cardiac autonomic function. Heart rhythm. 3 (12), 1428–1435. 10.1016/j.hrthm.2006.08.025 17161785

[B6] Bauer A. Kantelhardt J. W. Barthel P. Schneider R. Mäkikallio T. Ulm K. (2006). Deceleration capacity of heart rate as a predictor of mortality after myocardial infarction: Cohort study. Lancet 367 (9523), 1674–1681. 10.1016/S0140-6736(06)68735-7 16714188

[B7] Bibevski S. Dunlap M. E. (2004). Prevention of diminished parasympathetic control of the heart in experimental heart failure. Am. J. Physiol. Heart Circ. Physiol. 287 (4), H1780–H1785. 10.1152/ajpheart.00430.2004 15191889

[B8] Bibevski S. Zhou Y. McIntosh J. M. Zigmond R. E. Dunlap M. E. (2000). Functional nicotinic acetylcholine receptors that mediate ganglionic transmission in cardiac parasympathetic neurons. J. Neurosci. 20 (13), 5076–5082. 10.1523/JNEUROSCI.20-13-05076.2000 10864965 PMC6772271

[B9] Chen W. Liu Z. Xiao P. Xu Y. Li D. Xiong Q. (2022). Extracardiac vagal stimulation-assisted cardioneuroablation: Dynamically evaluating the impact of sequential ganglionated plexus ablation on vagal control of SAN and AVN in patients with sinoatrial node dysfunction. J. Cardiovasc Dev. Dis. 9 (6), 188. 10.3390/jcdd9060188 35735817 PMC9225033

[B10] Chiou C. W. Eble J. N. Zipes D. P. (1997). Efferent vagal innervation of the canine atria and sinus and atrioventricular nodes. The third fat pad. Circulation 95 (11), 2573–2584. 10.1161/01.cir.95.11.2573 9184589

[B11] Csepe T. A. Kalyanasundaram A. Hansen B. J. Zhao J. Fedorov V. V. (2015). Fibrosis: A structural modulator of sinoatrial node physiology and dysfunction. Front. Physiol. 6, 37. 10.3389/fphys.2015.00037 25729366 PMC4325882

[B12] de Marneffe M. Gregoire J. M. Waterschoot P. Kestemont M. P. (1993). The sinus node function: Normal and pathological. Eur. Heart J. 14 (5), 649–654. 10.1093/eurheartj/14.5.649 8508858

[B13] de Marneffe M. Jacobs P. Haardt R. Englert M. (1986). Variations of normal sinus node function in relation to age: Role of autonomic influence. Eur. Heart J. 7 (8), 662–672. 10.1093/oxfordjournals.eurheartj.a062120 3769951

[B14] Debruyne P. Rossenbacker T. Collienne C. Roosen J. Ector B. Janssens L. (2018). Unifocal right-sided ablation treatment for neurally mediated syncope and functional sinus node dysfunction under computed tomographic guidance. Circ. Arrhythm. Electrophysiol. 11 (9), e006604. 10.1161/CIRCEP.118.006604 30354289

[B15] Debruyne P. Rossenbacker T. Janssens L. Collienne C. Ector J. Haemers P. (2021). Durable physiological changes and decreased syncope burden 12 Months after unifocal right-sided ablation under computed tomographic guidance in patients with neurally mediated syncope or functional sinus node dysfunction. Circ. Arrhythm. Electrophysiol. 14 (6), e009747. 10.1161/CIRCEP.120.009747 33999698 PMC8208097

[B16] Dobrzynski H. Boyett M. R. Anderson R. H. (2007). New insights into pacemaker activity: Promoting understanding of sick sinus syndrome. Circulation 115 (14), 1921–1932. 10.1161/CIRCULATIONAHA.106.616011 17420362

[B17] Doyen B. Matelot D. Carré F. (2019). Asymptomatic bradycardia amongst endurance athletes. Phys. Sportsmed. 47 (3), 249–252. 10.1080/00913847.2019.1568769 30640577

[B18] Evrengul H. Tanriverdi H. Kose S. Amasyali B. Kilic A. Celik T. (2006). The relationship between heart rate recovery and heart rate variability in coronary artery disease. Ann. Noninvasive Electrocardiol. 11 (2), 154–162. 10.1111/j.1542-474X.2006.00097.x 16630090 PMC7313315

[B19] Glikson M. Nielsen J. C. Kronborg M. B. Michowitz Y. Auricchio A. Barbash I. M. (2021). 2021 ESC Guidelines on cardiac pacing and cardiac resynchronization therapy. Eur. Heart J. 42 (35), 3427–3520. 10.1093/eurheartj/ehab364 34455430

[B20] Hou Y. Scherlag B. J. Lin J. Zhou J. Song J. Zhang Y. (2007). Interactive atrial neural network: Determining the connections between ganglionated plexi. Heart rhythm. 4 (1), 56–63. 10.1016/j.hrthm.2006.09.020 17198991

[B21] Hu F. Zheng L. Liang E. Ding L. Wu L. Chen G. (2019). Right anterior ganglionated plexus: The primary target of cardioneuroablation? Heart rhythm. 16 (10), 1545–1551. 10.1016/j.hrthm.2019.07.018 31330187

[B22] Jensen P. N. Gronroos N. N. Chen L. Y. Folsom A. R. deFilippi C. Heckbert S. R. (2014). Incidence of and risk factors for sick sinus syndrome in the general population. J. Am. Coll. Cardiol. 64 (6), 531–538. 10.1016/j.jacc.2014.03.056 25104519 PMC4139053

[B23] Kusumoto F. M. Schoenfeld M. H. Barrett C. Edgerton J. R. Ellenbogen K. A. Gold M. R. (2019). 2018 ACC/AHA/HRS guideline on the Evaluation and management of Patients with bradycardia and Cardiac Conduction delay: A report of the American college of Cardiology/American heart association task force on clinical practice guidelines and the heart rhythm society. J. Am. Coll. Cardiol. 74 (7), e51–e156. 10.1016/j.jacc.2018.10.044 30412709

[B24] Lo L. W. Scherlag B. J. Chang H. Y. Lin Y. J. Chen S. A. Po S. S. (2013). Paradoxical long-term proarrhythmic effects after ablating the "head station" ganglionated plexi of the vagal innervation to the heart. Heart rhythm. 10 (5), 751–757. 10.1016/j.hrthm.2013.01.030 23357542

[B25] Lu C. S. Guo C. J. Fang D. P. Hao P. He D. F. Xu A. G. (2020). Initial experience with ablation of the innervation surrounding sinus and atrioventricular nodes to treat paroxysmal bradyarrhythmia. Chin. Med. J. Engl. 133 (2), 134–140. 10.1097/CM9.0000000000000595 31880742 PMC7028169

[B26] Malik M. Hnatkova K. Huikuri H. V. Lombardi F. Schmidt G. Zabel M. (2019). CrossTalk proposal: Heart rate variability is a valid measure of cardiac autonomic responsiveness. J. Physiol. 597 (10), 2595–2598. 10.1113/JP277500 31006862 PMC6826215

[B27] Mandel W. J. Hayakawa H. Allen H. N. Danzig R. Kermaier A. I. (1972). Assessment of sinus node function in patients with the sick sinus syndrome. Circulation 46 (4), 761–769. 10.1161/01.cir.46.4.761 5072776

[B28] Manoj P. Kim J. A. Kim S. Li T. Sewani M. Chelu M. G. (2022). Sinus node dysfunction: Current understanding and future directions. Am. J. Physiol. Heart Circ. Physiol. 324, H259–H278. 10.1152/ajpheart.00618.2022 36563014 PMC9886352

[B29] Manolis A. A. Manolis T. A. Apostolopoulos E. J. Apostolaki N. E. Melita H. Manolis A. S. (2021). The role of the autonomic nervous system in cardiac arrhythmias: The neuro-cardiac axis, more foe than friend? Trends Cardiovasc Med. 31 (5), 290–302. 10.1016/j.tcm.2020.04.011 32434043

[B30] Marcus B. Gillette P. C. Garson A. Jr (1991). Electrophysiologic evaluation of sinus node dysfunction in postoperative children and young adults utilizing combined autonomic blockade. Clin. Cardiol. 14 (1), 33–40. 10.1002/clc.4960140108 2019029

[B31] Marmerstein J. T. McCallum G. A. Durand D. M. (2021). Direct measurement of vagal tone in rats does not show correlation to HRV. Sci. Rep. 11 (1), 1210. 10.1038/s41598-020-79808-8 33441733 PMC7807082

[B32] Nielsen J. C. Thomsen P. E. Højberg S. Møller M. Vesterlund T. Dalsgaard D. (2011). A comparison of single-lead atrial pacing with dual-chamber pacing in sick sinus syndrome. Eur. Heart J. 32 (6), 686–696. 10.1093/eurheartj/ehr022 21300730

[B33] Pachon-M. J. C. Pachon-M. E. I. Pachon C. T. C. Santillana-P. T. G. Lobo T. J. Pachon-M. J. C. (2020). Long-term evaluation of the vagal denervation by cardioneuroablation using holter and heart rate variability. Circ. Arrhythm. Electrophysiol. 13 (12), e008703. 10.1161/CIRCEP.120.008703 33198486

[B34] Pachon J. C. Pachon E. I. Cunha Pachon M. Z. Lobo T. J. Pachon J. C. Santillana T. G. (2011). Catheter ablation of severe neurally meditated reflex (neurocardiogenic or vasovagal) syncope: Cardioneuroablation long-term results. Europace 13 (9), 1231–1242. 10.1093/europace/eur163 21712276

[B35] Pachon J. C. Pachon E. I. Pachon J. C. Lobo T. J. Pachon M. Z. Vargas R. N. (2005). Cardioneuroablation"-new treatment for neurocardiogenic syncope, functional AV block and sinus dysfunction using catheter RF-ablation. Europace 7 (1), 1–13. 10.1016/j.eupc.2004.10.003 15670960

[B36] Pick A. Langendorf R. Katz L. N. (1951). Depression of cardiac pacemakers by premature impulses. Am. Heart J. 41 (1), 49–57. 10.1016/0002-8703(51)90005-1 14799438

[B37] Qin M. Zhang Y. Liu X. Jiang W. F. Wu S. H. Po S. (2017). Atrial ganglionated plexus modification: A novel approach to treat symptomatic sinus bradycardia. JACC Clin. Electrophysiol. 3 (9), 950–959. 10.1016/j.jacep.2017.01.022 29759719

[B38] Randall W. C. Ardell J. L. Wurster R. D. Milosavljevic M. (1987). Vagal postganglionic innervation of the canine sinoatrial node. J. Auton. Nerv. Syst. 20 (1), 13–23. 10.1016/0165-1838(87)90077-4 3655182

[B39] Rocchetti M. Malfatto G. Lombardi F. Zaza A. (2000). Role of the input/output relation of sinoatrial myocytes in cholinergic modulation of heart rate variability. J. Cardiovasc Electrophysiol. 11 (5), 522–530. 10.1111/j.1540-8167.2000.tb00005.x 10826931

[B40] Sassi R. Cerutti S. Lombardi F. Malik M. Huikuri H. V. Peng C. K. (2015). Advances in heart rate variability signal analysis: Joint position statement by the e-cardiology ESC working group and the European heart rhythm association co-endorsed by the asia pacific heart rhythm society. Europace 17 (9), 1341–1353. 10.1093/europace/euv015 26177817

[B41] Sathnur N. Ebin E. Benditt D. G. (2021). Sinus node dysfunction. Card. Electrophysiol. Clin. 13 (4), 641–659. 10.1016/j.ccep.2021.06.006 34689892

[B42] Sheldon R. S. Grubb B. P. 2nd Olshansky B. Shen W. K. Calkins H. Brignole M. (2015). 2015 heart rhythm society expert consensus statement on the diagnosis and treatment of postural tachycardia syndrome, inappropriate sinus tachycardia, and vasovagal syncope. Heart rhythm. 12 (6), e41–e63. 10.1016/j.hrthm.2015.03.029 25980576 PMC5267948

[B44] Shivkumar K. Ajijola O. A. Anand I. Armour J. A. Chen P. S. Esler M. (2016). Clinical neurocardiology defining the value of neuroscience-based cardiovascular therapeutics. J. Physiol. 594 (14), 3911–3954. 10.1113/JP271870 27114333 PMC4945719

[B45] Szatmáry L. Jouve A. Pinot J. J. Torresani J. (1983). Comparative study of electrophysiological and Holter monitoring data in estimating sinoatrial function. Significance of intrinsic heart rate in disclosing autonomic sinus node dysfunction. Cardiology 70 (4), 184–193. 10.1159/000173592 6640559

[B46] Tu B. Wu L. Hu F. Fan S. Liu S. Liu L. (2022). Cardiac deceleration capacity as an indicator for cardioneuroablation in patients with refractory vasovagal syncope. Heart rhythm. 19 (4), 562–569. 10.1016/j.hrthm.2021.12.007 34896621

[B47] Vandenberk B. Lei L. Y. Ballantyne B. Vickers D. Liang Z. Sheldon R. S. (2022). Cardioneuroablation for vasovagal syncope: A systematic review and meta-analysis. Heart rhythm. 19 (22), 180402088–180418124. 10.1016/j.hrthm.2022.06.017 35716859

[B48] Vavetsi S. Nikolaou N. Tsarouhas K. Lymperopoulos G. Kouzanidis I. Kafantaris I. (2008). Consecutive administration of atropine and isoproterenol for the evaluation of asymptomatic sinus bradycardia. Europace 10 (10), 1176–1181. 10.1093/europace/eun211 18701603

[B43] Writing Committee Members Shen W. K. Sheldon R. S. Benditt D. G. Cohen M. I. Forman D. E. Goldberger Z. D. (2017). 2017 ACC/AHA/HRS guideline for the evaluation and management of patients with syncope: A report of the American college of Cardiology/American heart association task force on clinical practice guidelines and the heart rhythm society. Heart rhythm. 14 (8), e155–e217. 10.1016/j.hrthm.2017.03.004 28286247

[B49] Yao Y. Shi R. Wong T. Zheng L. Chen W. Yang L. (2012). Endocardial autonomic denervation of the left atrium to treat vasovagal syncope: An early experience in humans. Circ. Arrhythm. Electrophysiol. 5 (2), 279–286. 10.1161/CIRCEP.111.966465 22275485

[B50] Zaza A. Lombardi F. (2001). Autonomic indexes based on the analysis of heart rate variability: A view from the sinus node. Cardiovasc Res. 50 (3), 434–442. 10.1016/s0008-6363(01)00240-1 11376619

[B51] Zhao L. Jiang W. Zhou L. Wang Y. Zhang X. Wu S. (2015). Atrial autonomic denervation for the treatment of long-standing symptomatic sinus bradycardia in non-elderly patients. J. Interv. Card. Electrophysiol. 43 (2), 151–159. 10.1007/s10840-015-9981-8 25693516

[B52] Zheng L. Sun W. Liu S. Liang E. Du Z. Guo J. (2020). The diagnostic value of cardiac deceleration capacity in vasovagal syncope. Circ. Arrhythm. Electrophysiol. 13 (12), e008659. 10.1161/CIRCEP.120.008659 33197331

